# Risk Factors Associated with Prevalence of *Candida albicans*, *Gardnerella vaginalis*, and *Trichomonas vaginalis* among Women at the District Hospital of Dschang, West Region, Cameroon

**DOI:** 10.1155/2020/8841709

**Published:** 2020-08-05

**Authors:** Vincent Khan Payne, Tsonang Tassongwa Florence Cécile, Yamssi Cedric, Noumedem Anangmo Christelle Nadia, Ouaba José

**Affiliations:** ^1^Department of Animal Biology, Faculty of Science, University of Dschang, P.O. Box 067, Dschang, Cameroon; ^2^Department of Biomedical Sciences, Faculty of Health Sciences, University of Bamenda, P.O. Box 39, Bambili, Cameroon; ^3^Department of Microbiology, Hematology and Immunology Faculty of Medicine and Pharmaceutical Sciences, University of Dschang, P.O. Box 96, Dschang, Cameroon

## Abstract

**Background:**

Vaginal or genitourinary infections are a major cause of morbidity, sterility, and increase in the vulnerability to cancers and HIV/AIDS infection. The aim of this study was to determine the prevalence of vaginal infections of *C. albicans*, *G. vaginalis*, and *T. vaginalis* among women in the locality of Dschang, West Region of Cameroon.

**Method:**

A prospective study was carried out in the District Hospital of Dschang. After obtaining informed consent, one thousand and one (1001) samples of vaginal swabs were collected. Biological diagnosis was carried out on fresh samples, Gram stained, and then cultivated in Sabouraud agar in a Petri dish, in order to isolate and identify the various infectious agents.

**Results:**

Five hundred and twenty-five (525) women were diagnosed positive, hosting at least one of these microorganisms, making an overall prevalence of 52.44%. Two hundred and fifty-six (256) women (25.57%) were infected with *C. albicans*, and 171 (17.08%) with *G. vaginalis.* Ninety-five (9.49%) were infected with both *C. albicans* and *G. vaginalis*, 2 (0.20%) with *C. albicans* and *T. vaginalis*, and 1 (0.1%) with *G. vaginalis* and *T. vaginalis*.

**Conclusion:**

Drastic measures should be taken in order to improve life styles to regress the frequency of these infections. Results obtained in this study, will help to educate and shed more light on the prevalence of vaginal infections in the West Region of Cameroon.

## 1. Introduction

Vaginal organ infections have an important socioeconomic impact in developing countries in general and in Cameroon in particular. They represent one of the major sources of morbidity and sterility and increase the exposure to cancers and HIV/AIDS infection [1]. Vaginitis and vaginosis are the most important among infectious diseases found in medical centres today. There are many sources of morbidities (sterility, abortions, and premature deliveries) among women of productivity age [2].

*Trichomonas vaginalis, Gardnerella vaginalis*, and *Candida albicans* are responsible for 90% of vaginitis in Iran [1]. Vaginitis is a local inflammatory process taking place at the level of the vaginal cavity, which is due to the presence of one or more infectious agents (bacteria, parasites, fungi, etc.). Vaginitis can either be specific or nonspecific. For specific vaginitis, the pathogenic agent in many cases has exogenous origin. The reaction is made visible by a purulent discharge in which the pathogenic agent can be identified. Most of the time, vaginitis are caused by *Trichomonas vaginalis* or *Candida albicans*, but many other microorganisms, such as commensal microorganisms (*Lactobacillus* or Gram + *Bacillus*) can bring about the same kind of pathology in certain cases [3]. Bacterial vaginosis (normally without inflammation) comes from an imbalance of the vaginal flora with the shift of lactobacilli by anaerobic commensal microorganisms. This disequilibrium leads to the loss of lactobacilli and to a change of vaginal pH.

Bacterial vaginosis is more frequent in sexually active women. This vaginosis can either be symptomatic or asymptomatic [4]. Genital infections have become a huge health problem in Cameroon with devastating consequences. Premature deaths, destabilization of the family, increased number of orphans, increased medical and funeral expenses, increased absenteeism from work, decreased productivity and massive loss of jobs, reduction of qualified manpower, and increased misery and poverty are very glaring. Studies on vagina infection have been conducted in several countries [2], but there is insufficient data from Cameroon, with regard to prevalence and associated risk factors among women. There is a dearth of knowledge on the aetiology of genital infections in Dschang. Thus, in line with global prevention efforts, the present investigation was undertaken to determine the risk factors associated with prevalence of *Candida albicans*, *Gardnerella vaginalis*, and *Trichomonas vaginalis* among women of reproductive age at the District Hospital of Dschang.

## 2. Materials and Methods

### 2.1. Presentation of the Area of Study

This study was cross-sectional and was conducted in the District Hospital of Dschang in 2018, targeting women coming for consultation. It received daily about 40 women from different sociocultural backgrounds for consultation.

### 2.2. Sampling and Target Population

Our sample was made up of 1001 women between 15 and 55 years of age, who manifested, or not, the symptoms of vaginal infections. Women on imidazole antibiotics and antifungal were excluded from the study, as well as those who had sexual intercourse a day before or who did their personal hygiene (douching) and those in their menstrual period. The questionnaire given to these patients helped us to identify the risk factors. Such data included the age, area of residence, religion, tribe, marital status, type of marriage, occupation and that of spouse, the number of sexual partners, whether or not a patient has suffered from a genital infection in the past, and the presence or absence of discharge.

#### 2.2.1. Swab

Cervicovaginal swabs were collected from each woman through insertion of a sterile speculum into the posterior side wall of the vagina. The individual was requested to lie down in gynaecological position on a bed previously disinfected, with the help of two swabs, one of which allowed us to take secretion samples at the level of the cervix and the second one on the vaginal wall [5].

The macroscopic observation of the vaginal organ helped us to detect some abnormalities on the vagina and also to appreciate the colour and the leucorrhoea consistency, and finally a drop of secretion was placed on a NaOH solution at 10%. Microscopic observations were done by a direct microscopy, Gram staining, and finally culture using Petri dishes.

#### 2.2.2. Fresh State and *T. vaginalis* Identification

*T. vaginalis* was identified by morphological characteristics as well as the mobility as described by Cheesbrough [6].

#### 2.2.3. Identification of *Gardnerella vaginalis*

A current international consensus establishes the diagnosis of *Gardnerella* when three of the following criteria are met [3]:

- Vaginal pH greater than 4, 5.
- Positive test of NaOH.
- Presence of clue cells in microscopic examination.
- Malodorous and adherent leucorrhoea [4]

#### 2.2.4. Isolation and Identification of *Candida albicans*

Sabouraud agar medium was used for isolation and the filamentation test for identification, according to Catalan et al. [3].

### 2.3. Parameters Studied

#### 2.3.1. Prevalence

The prevalence (*P*) was calculated using the formula

(1) $$\begin{array}{c} P=\frac{\text{number of individuals infected}}{\text{number of individuals examined}}\times 100, \end{array}$$

where *P* is prevalence.

#### 2.3.2. Statistical Analysis

The results were analysed with the statistical analysis program R.

## 3. Results

Three types of microorganisms, causative agents of vaginitis, and vaginosis were identified. Of 1001 samples collected, 525 patients were positive for at least one microorganism, together making up a total prevalence of 52, 44%. Tables 1–4 represent the prevalence of vaginal infections with respect to some risk factors. It appears from these tables that the prevalence of *C. albicans* is systematically higher than *G. vaginalis*. The population mostly affected (Table 1) by *G. vaginalis* were of the age range, 15 to 25 years (18.07%), followed by those of age range from 36 to 45 years (17.91%), whereas those mostly affected by *C. albicans* were of age range from 26 to 35 years (40.60%).

Table 2 shows the prevalence in symptomatic and asymptomatic women. It appears from this table that *C. albicans* and *G. vaginalis* presented prevalence of 68.66% and 47.21%, in symptomatic women, respectively. Nevertheless, 24.86% (*C. albicans*) and 20.31% (*G. vaginalis*) were asymptomatic women.

The study involved 768 single women (nonvirgin) and 233 married women of whom 175 were involved in monogamous marital relationship and 58 in polygamous marital relationship (Table 3). Married women from monogamous marriages were the most infected by *C. albicans* (31.43%), while those from polygamous marriages were mostly infected by *G. vaginalis* (18.97%).

Single women are more infected by *G. vaginalis* (17.62%) than virgin women (2.78%). Likewise, virgin women showed low prevalence (11%) of *C. albicans*, compared to single women (18%).

Table 4 shows that the prevalence of *C. albicans* (22.51%) is greater than *G. vaginalis* (5.63%) in women using contraceptives (condom). Nevertheless, it is even higher (56.52%) among those using pills.

Concerning personal hygiene (Table 5), women who did their vaginal douching were more exposed to *G. vaginalis* (28.66%) than *C. albicans* (23.67%) contrary to those who did not do it at all (12.13% and 26.39%, respectively), and concerning all the other factors, the prevalence of *C. albicans* was higher than that of *G. vaginalis.* Women using undergarments made from synthetic materials were more infected than those using cotton underwear. For these first two cases, the prevalence of *C. albicans* was indeed higher than *G. vaginalis*.

## 4. Discussion

Higher prevalence of vaginal infection in all the age groups could be due to the fact that women are more sexually active and some of them have many sexual partners, whether they are married or not, in addition to the poor hygiene conditions. These results are in accordance with those of Kamga et al. [7] and Achondou et al. [8] in Cameroon.

The high prevalence of these infections could be due to the anatomy of the female vaginal organ which favours penetration and the installation of the infection and poor personal hygiene. In fact, poor personal hygiene contributes to the change in pH from its normal state of acidity (3) to 3.5 [6], which could lead to the alteration of the normal flora; tight and synthetic underwear (in nylon) prevent aeration of the vagina, produce heat in the vaginal region, and then favour the proliferation of microorganisms. It is also due to unprotected sexual practices: for some of them, unprotected sexual intercourse with a healthy man can cause less harm. This vaginosis develops because the sperm, too alkaline, reduces acidity in the vaginal milieu. However, infected men naturally transmit the microorganisms to their partner due to the anatomy of female vaginal organ which acts like a reservoir. That is why 100% of women are generally infected by *T. vaginalis* when their partner is infected too [9].

The transmission of *T. vaginalis* and *G. vaginalis* is mainly sexual. But a nonsexual transmission can be evoked by the fact that microorganisms remain alive on bedding and toilette seats, as well as into bathing water [9]. It is possible with that to explain the cases of infection by *T. vaginalis* and *G. vaginalis* among virgin women.

The prevalence of *G. vaginalis* (26.67%) is higher than that reported by Fusi-Ngwa et al. [10] in the same locality (1.6%). It is still higher than that reported in other localities. In Nigeria, Azargoon and Darwshzadeh [11] had reported 16.00% among pregnant women. In Brazil, Murta et al. [12] reported 23.6% of prevalence of *G. vaginalis* among women.

This prevalence (26.67%) is similar to that reported by Nwadioha et al. [13] among women at Kano Hospital (Aminu Kano) in Nigeria, which was 26.05%, and to that reported by Rao et al. [14] among rural women in India. However, this prevalence remains lower, compared to the 86% reported by Fernández-Limia et al. [15] and to the 86% reported by Oyewole et al. [16] among women in Sagamu, Ogun State in Nigeria. Differences in prevalence reported in different settings could be due to environmental, behavioral, and socioeconomic status and stressor differences with geographical variation.

The prevalence of *C. albicans* in our study was 35.26%. These results are higher than those reported by Kalantari et al. [2], Adeolu et al. [4], Ángel-müller et al. [17], Haltas et al. [18], and Fusi-Ngwa et al. [10], but lower than those reported by Chervenkova et al. [19] and Mathew et al. [20]. However, they remain between the range of 17 to 39% reported by Nyirjesy [21]. The high prevalence of *G. vaginalis* and *C. albicans* among patients living with vaginal infections confirms the fact that these microorganisms play an important role in the pathogenesis. The pathogenesis of these infections is very complex and has not been well elucidated. Nevertheless, it is evident that certain factors such as adenosine, cytotoxins, enzymes of *G. vaginalis* and *C. albicans*, and certain anaerobic bacteria impact this process [3].

Pregnancy causes hormonal variations which deteriorate the metabolism, increase the rate of glucose, affect vaginal pH, weaken the immune system, and make the pregnant women more susceptible to infections [22]. It is certainly what could explain the increase in *C. albicans* infections among pregnant women (31.19%) over nonpregnant women (24.16%). On the other hand, the prevalence of *G. vaginalis* is low in pregnant women. This can be due to the fact that they are not very much sexually active during this period [23].

Two hundred and thirty-three (23.28%) of the patients were symptomatic, that is, 23.28% of the whole sample. Among them, 12.02% had no pathogen. In this case, an investigation of organisms, which grow slowly or with difficultly, is necessary to know the origin of the disease. It can also be due to the fact that many of these patients make their personal hygiene using various types of chemical products (disinfectants) and inappropriate soaps to wash their vaginal organs [24].

Foul-smelling leucorrhoea, vaginal itches, and vaginitis are the most frequent complaints among women in the age of procreation in many countries in the world. Most of the women living with sexually transmitted diseases (STDs) have vaginitis and frequent vaginal discharges [4]. In this study, we received 233 symptomatic patients (87.98%) presenting at least one germ and 768 asymptomatic patients (41.28%) presenting at least one germ. 12.02% of the symptomatic patients presented recurrent symptoms of nonspecific vaginitis.

The prevalence of multiple infections was lower in this study than that reported by others [7, 8]. However, *G. vaginalis* was frequently associated with *C. albicans* (9.49%). These results are similar to those reported by Shey Nsagha et al. [23]. This could justify the fact that *G. vaginalis* and *C. albicans* were not located at the same area. *G. vaginalis* is usually located at the level of the vagina normal flora, whereas *C. albicans* is generally found at the level of the vaginal wall, and also they do not use the same resources. Triple infections were totally absent in our study.

Sexually active women in this study were 511 (52.96%) infected. This could be explained by the fact that many among them have many sexual partners and do not use condoms, in addition to perhaps very poor personal hygiene. However, the frequency of these infections (36.11% of *C. albicans*, and 2.78% of *G.*) among virgin women is also important. These results are similar to those of Richoz [9]. This could be explained by the fact that they have not yet been taught how to or mastered the basic rudiments of douching; they use joint toilettes and co-use towels, underwear, and so on.

Use of the pill generated a prevalence of 80.43%. In fact, contraceptive pills are made up of progestative and oestrogenic hormones that are antagonists in structure and in function with progesterone and oestrogen. It inhibits the synthesis of oestrogens, deteriorates the metabolism of glucose, and affects the vaginal pH, making the individual concerned to become more vulnerable to infections [3].

## 5. Conclusion

Vaginal organ infections are now gaining greater recognition as an important source of reproductive problems. Determining the aetiology of these patients and their risk factors is important to establish the treatment strategy in the healthcare units. We therefore recommend that women should be routinely screened for vagina infections to reduce complications related to these infections.

## Figures and Tables

**Table 1:** Prevalence as a function of age.

	Agents	15–25 (*n* = 653)		26–35 (*n* = 266)		36–45 (*n* = 67)		46–55 (*n* = 15)		Total no. examined
		NE	P (%)	NE	P (%)	NE	P (%)	NE	P (%)	
Single infections	*C. albicans*	156	23.89	78	29.32	18	26.87	4	26.67	256
	*G. vaginalis*	118	18.07	39	14.66	12	17.9	2	13.33	171
	*T. vaginalis*	0	0	0	0	0	0	0	0	0
Multiple infections	*C. albicans* and *G. vaginalis*	58	8.88	30	11.28	7	10.45	0	0	95
	*C. albicans* and *T. vaginalis*	1	0.15	1	0.38	0	0	0	0	2
	*G. vaginalis* and *T. vaginalis*	1	0.15	0	0	0	0	0	0	1
	*C. albicans, G. vaginalis*, and *T. vaginalis*	0	0	0	0	0	0	0	0	0
Total		334		148		37		6		525

*C*: *Candida*, *G*: *Gardnerella*, *T*: *Trichomonas*, NE: number examined, and P: prevalence.

**Table 2:** Prevalence in symptomatic and asymptomatic women.

	Infectious agents	Symptomatic (*n* = 233)		Asymptomatic (*n* = 768)		Total
		NE	P (%)	NE	P (%)	
Single infections	*C. albicans*	160	68.66	191	24.86	351
	*G. vaginalis*	110	47.21	156	20.31	266
	*T. vaginalis*	0	0	0	0	0
Multiple infections	*C. albicans* and *G. vaginalis*	65	27.90	30	3.91	95
	*C. albicans* and *T. vaginalis*	0	0	2	0	2
	*G. vaginalis* and *T. vaginalis*	0	0	1	0	1
	*C. albicans, G. vaginalis*, and *T. vaginalis*	0	0	0	0	0

**Table 3:** Prevalence as function of status and marital regime.

	Infectious agents	MM (*n* = 175)		MP (*n* = 58)		Singles (*n* = 768)		Virgin (36)		Total
		NE	P (%)	NE	P (%)	NE	P (%)	NE	P (%)	
Single infections	*C. albicans*	55	31.43	14	24.14	187	24.35	13	36.11	256
	*G. vaginalis*	25	14.29	11	18.97	135	17.58	1	2.77	171
	*T. vaginalis*	0	0	0	0	0		0	0	
Multiples infections	*C. albicans* and *G. vaginalis*	18	10.07	7	12.07	70	9, 11	0	0	95
	*C. albicans* and *T. vaginalis*	0	0	0	0	2	2, 60	0	0	2
	*G. vaginalis* and *T. vaginalis*	0	0	0	0	1	1, 30	0	0	1
	*C. albicans, G. vaginalis*, and *T. vaginalis*	0	0	0	0	0	0	0	0	0
Total		98	55.79	32	55.18	395	54.94	14	38.88	525

MM: married monogamous; MP: married polygamous.

**Table 4:** Prevalence as function of contraceptive method.

	Infectious agents	Condom (*n* = 391)		Pills (*n* = 46)		No contraceptive (*n* = 564)		Total
		NE	P (%)	NE	P (%)	NE	P (%)	
Single infections	*C. albicans*	88	22.51	26	56.52	142	25.18	256
	*G. vaginalis*	22	5.63	6	13.04	143	25.35	171
	*T. vaginalis*	0	0	0	0	0	0	0
Multiple infections	*C. albicans* and *G. vaginalis*	11	2.81	5	10.70	79	14.01	95
	*C. albicans* and *T. vaginalis*	0	0	0	0	2	0.35	2
	*G. vaginalis* and *T. vaginalis*	0	0	0	0	1	0.18	1
Total		121	30.94	37	80.43	367	65.07	525

**Table 5:** Prevalence as function of WC type, underwear, and vaginal douching.

Infectious agents		WC				Type of underwear				Vaginal douching				Total
		Pot (*n* = 311)		Hole (traditional) (*n* = 565)		Synt (*n* = 149)		Cotton (*n* = 852)		Yes (*n* = 300)		No (*n* = 701)		
		NE	P (%)	NE	P (%)	NE	P (%)	NE	P (%)	NE	P (%)	NE	P (%)	
Single infections	*C. albicans*	88	28.29	136	43.73	47	31.54	209	24.53	71	23.67	185	26.39	256
	*G. vaginalis*	57	18.33	97	31.19	30	20.13	141	16.55	86	28.66	85	12.13	171
	*T. vaginalis*	0	0	0	0	0	0	0	0	0	0	0	0	0
Multiple infections	*C. albicans* and *G. vaginalis*	25	8.04	53	17.04	24	16.11	71	8.33	54	18.00	41	5.85	95
	*C. albicans* and *T. vaginalis*	0	0	2	0.64	0	0	2	0.23	0	0	2	0.28	2
	*G. vaginalis* and *T. vaginalis*	1	0.32	0	0	0	0	1	0	0	0	1	0.14	1
Total		171	54.98	288	50.97	101	67.79	421	48.41	211	70.33	314	44.79	525

*C*: *Candida*, *G*: *Gardnerella*, *T*: *Trichomonas*, NE: number examined, P: prevalence, and Synt: synthetic.

## Data Availability

Data and material are available to other researchers upon request.

## Abbreviations

*C. albicans*: *Candida albicans*
*G. vaginalis*: *Gardnerella vaginalis*
*T. vaginalis*: *Trichomonas vaginalis.*
